# Biodegradable polylactic acid emulsion ink based on carbon nanotubes and silver for printed pressure sensors

**DOI:** 10.1038/s41598-024-60315-z

**Published:** 2024-05-14

**Authors:** Maedeh Najafi, Emilie Forestier, Milad Safarpour, Luca Ceseracciu, Arkadiusz Zych, Ahmad Bagheri, Laura Bertolacci, Athanassia Athanassiou, Ilker Bayer

**Affiliations:** 1 Smart Materials, Istituto Italiano di Tecnologia, Via Morego 30, 16163 Genoa, Italy; 2 iCub Tech, Istituto Italiano di Tecnologia, Via S. Quirico 9d, 16163 Genoa, Italy; 3 Materials Characterization, Istituto Italiano di Tecnologia, Via Morego 30, 16163 Genoa, Italy; 4 Graphene Labs, Istituto Italiano di Tecnologia, Via Morego 30, 16163 Genoa, Italy

**Keywords:** Green electronics, Biodegradable ink, Polylactic acid, Emulsion binder system, Hybrid fillers, Environmental impact, Biomaterials, Bionanoelectronics, Biosensors, Electrical and electronic engineering

## Abstract

Investigating biodegradable and biocompatible materials for electronic applications can lead to tangible outcomes such as developing green-electronic devices and reducing the amount of e-waste. The proposed emulsion-based conducting ink formulation takes into consideration circular economy and green principles throughout the entire process, from the selection of materials to the production process. The ink is formulated using the biopolymer polylactic acid dissolved in a sustainable solvent mixed with water, along with conductive carbon nanotubes (CNTs) and silver flakes as fillers. Hybrid conductive fillers can lower the percolation threshold of the ink and the production costs, while maintaining excellent electrical properties. The coating formed after the deposition of the ink, undergoes isothermal treatment at different temperatures and durations to improve its adhesion and electrical properties. The coating’s performance was evaluated by creating an eight-finger interdigitated sensor using a Voltera PCB printer. The sensor demonstrates exceptional performance when exposed to various loading and unloading pressures within the 0.2–500.0 kPa range. The results show a consistent correlation between the change in electrical resistance and the stress caused by the applied load. The ink is biodegradable in marine environments, which helps avoiding its accumulation in the ecosystem over time.

## Introduction

The widespread use of sensors in healthcare, manufacturing, and other control systems is facilitating the path toward a modern, safe, and interconnected society. However, factors including high production costs, inadequate sensitivity, rigid and expensive materials have so far limited their wide acceptance in industries like wearable electronics, health monitoring, and soft robotics^1,2^. Functionalizing flexible substrates with conductive inks can provide a sustainable and versatile platform for manufacturing electronic devices. This approach offers several advantages, such as the ability to design green flexible conductors^3,4^. Conductive inks have the ability to be printed on a range of flexible substrates, including plastics, paper, and textiles. This allows for the development of flexible electronic devices that can be bent, folded, and stretched without sustaining damage. The emergence of this technology presents exciting opportunities for wearable devices, medical sensors, and smart packaging. Printing using conductive inks is a more straightforward and more economical alternative to conventional manufacturing techniques, like lithography or etching. Conductive inks can be efficiently printed using various techniques such as screen printing, inkjet printing, or spray coating. Reducing the time and resources needed for manufacturing makes it possible to accelerate product development and commercialization^5,6^. Recent advancements in printed electronics, while considering the aforementioned benefits of conductive inks, have led to the formulation of long-term goals that involve the creation of interesting conductive inks with excellent electrical conductivity using recycled and/or bio-based materials. These components can be studied to fabricate a diverse range of flexible and stretchable electronic devices^7,8^.

Polylactic acid (PLA) has the highest production volumes nowadays, among the biobased and biodegradable bioplastics, which stand as the most promising sustainable alternative to conventional plastics^9,10^. Its amorphous section is susceptible to breakdown by hydrolysis and enzymatic activity, primarily because of its permeability to water. On the other hand, it can be brittle, a property that limits its applications, especially in fields like flexible electronics. From this perspective, the utilization of bio-derived polyurethane dispersions (PUDs) may improve the ductility of PLA, thus making it appropriate for use in scenarios that necessitate tactile flexibility. For this reason, in this study we used the combination of PLA with PUDs as matrix for the development of conductive inks for flexible electronic devices. On the top, PUDs were found to increase the hydrophilicity of PLA, and as a consequence to accelerate its biodegradation in sea water. The conductive fillers incorporated in the PLA/PUDs inks were silver flakes and carbon based nanofillers like carbon nanotubes (CNTs) or graphene nanoplatelets (GNPs).

In conductive inks, the material that conducts electricity is regarded as the most essential ingredient^11–13^. The selection of conductive materials is determined by parameters as adherence of the ink to the substrate, compatibility with the printing procedure^14^, etc. While keeping the ink’s high electrical conductivity is a priority, it is preferable to use a small amount of metallic fillers^15^. Therefore, increasing the electrical conductivity of the ink at a given concentration necessitates an optimal distribution of nanofillers. To achieve better conductivities at similar nanofiller loadings, it has been shown that a heterogeneous distribution is preferable to a homogeneous distribution across the matrix^16,17^. In order to achieve this goal, it was shown that utilizing approaches such as double percolation in hybrid systems with two different fillers, confining nanofillers in any one of the phases of a biphasic polymer, and creating repulsive forces between nanofillers and the host polymer were all effective ways to increase the ink’s conductivity^18^.

Carbon-based nanomaterials offer enormous promise for printed and flexible electronics usage, increasing their attractiveness^19–21^. They are highly appealing due to their structure, low weight, high aspect ratio, electrical conductivity, mechanical toughness, and cost-effectiveness. These materials’ wide range of features makes them suitable for various applications such as energy harvesting, electronic skin, and reinforced plastic materials^22^. GNPs, which are hybrids of graphene and graphite, are already available for industrial use. On the other hand, CNTs are electrically heterogeneous, which is one of the primary reasons they have garnered such a significant amount of interest^23^. However, the stability of CNT dispersion in water is still a big challenge because they frequently appear to agglomerate rapidly due to the strong van der Waals attraction they possess^24,25^.

Silver-based nanomaterials such as nanoparticles, micro and nanoflakes, and nanospheres show great potential for flexible and stretchable electronics. Silver has the highest conductivity among all available conductive fillers, making it ideal for electronic devices that require optimal performance with high conductivity^26,27^. In addition, silver-based materials not only have superior electrical properties but also exhibit excellent mechanical properties, including high ductility and tensile strength. These properties make them ideal for use in flexible and stretchable electronic devices. These materials can endure deformation and bending without affecting their conductivity, making them a perfect fit for wearable and implantable electronics^28,29^. Silver-based conductive ink is currently the top choice for printed electronics among the various nanoparticle-based conductive inks being studied. It offers superior oxidation resistance, electrical conductivity, and other important physical properties that ensure strong substrate adherence^30,31^. The fundamental issue with silver is the need for high-temperature annealing of the printed film to achieve exceptional conductivity^32,33^. At high annealing temperatures, the plastic and paper substrates are prone to degradation, a factor that restricts the application of fully silver-based inks in flexible electronics^33–35^. Hence, to keep costs down and avoid high processing temperatures, conductive inks with hybrid fillers, containing some amounts silver are a great alternative^7,36–38^.

Previous research on inks composed of carbon nanofillers, i.e., graphene and CNTs, silver, and a combination of both is presented in Table 1. Most CNT and silver-based conductive inks were produced using toxic solvents such as chloroform and DMF that are hazardous to humans and the environment. In addition, it is preferable to anneal silver at a high temperature of 200 °C to improve its electrical conductivity.

For the above reasons, the current research is focused on reducing the reliance on toxic organic solvents and substituting them with eco-friendly alternatives, such as water, in the development of conductive ink formulations. Therefore, the selection of suitable, environmentally sustainable solvents played a pivotal role in this study. Our objective is to contribute to the progress of sustainable practices in the field of printed electronics. Furthermore, our research is in line with current developments in additive manufacturing (AM) and soft robotics, which focus on incorporating sensors and actuators into 3D-printed structures to provide immediate feedback and improved functionality. This is consistent with the larger pattern of using multi-material extrusion to produce intelligent structures that have sensors embedded within them, as demonstrated by recent publications^39–41^. Through the development of eco-friendly ink formulations and the incorporation of sensors and actuators into 3D-printed structures, our research is leading the way in advancing additive manufacturing technologies towards more sustainable and efficient solutions.

**Table 1:** Literature on conductive inks based on conductive fillers.

Inks	Solvent	Printing method	Substrate	Annealing condition	Rs or σ	Ref.
MWCNTs/GelMA/DNA	Water	Screen printing	Paper, hydrogels, elastomers	–	24 ± 1.8 S/cm	^42^
MWCNTs	lysozyme solution (acidic buffer)	Pen direct writing	Glass, PET, silicon	Mild condition	500–1500 Ω/sq	^43^
AgNPs/graphene	DMF:EG: G	Inkjet	Polyvinyl alcohol	80 °C—10 to 30 min	0.3545 to 2.1517 S/m	^37^
AgNPs/CNTs	Chloroform and DMF	Free-standing composite	–	Mild condition	1228 S/cm	^20^
AgNPs/graphene	DMF	Inkjet	PI	400°C—30 min	20 ± 1 Ω/sq	^44^
AgNPs/graphene	Ethanol and acetone	Inkjet	PET	–	4.74 Ω/sq	^45^
Ag flakes/CNTs	Emulsion system	Spray coating	Paper/cotton fabric/nitrile gloves	110° C—120 min	300 Ω/sq	Current work

## Materials and methods

### Chemicals and materials

Amorphous PLA pellets (Ingeo Biopolymer 6060D) with a density of 1.24 g/cm^3^ and a glass transition temperature (Tg) of 55–60 °C were used as received from Nature Works (USA). Waterborne polyurethane (ROLFLEX BIO 49) was kindly provided by Lamberti S.p.A. in Varese, Italy, with a relative density of 0.907 ± 0.012 g/cm^3^ calculated from a dried film. Multi-walled carbon nanotubes (MWCNTs) were bought from Merck Life Science S.r.l. Milan, Italy, with a relative density of 2.1 g/cm^3^, diameter in the range of 110–170 nm, length in the range of 5–9 μm and a carbon content of 90 wt%. Merck Life Science S.r.l. in Milan, Italy, supplied the Silver Flakes 99.9% trace metal basis with an average particle size of 10 μm and a density of 10.49 g/cm^3^. GNPs aggregates were acquired through STREM Chemicals Inc. (USA). The platelets exhibit an average thickness ranging from 6 to 8 nm, a platelet width of 25 μm, a surface area of approximately 500 m^2^/g, a bulk density ranging from 0.03 to 0.10 g/cm^3^, an oxygen content of less than 1%, and a carbon content of at least 99 wt%. Ethyl acetate (EtOAc) was purchased from Merck Life Science S.r.l. in Milan, Italy, and was used as a solvent for making the polymer solution with 99.5% purity. Polyethylene glycol sorbitan monooleate (Tween 80) and sorbitane monooleate (Span 80), used as non-ionic surfactants, were purchased from Merck Life Science S.r.l. in Milan, Italy. Ultrapure Milli-Q water was used in making emulsion-based ink.

### Ink formulation

The formulation process for bio-based ink used in spray coating involves a sequence of straightforward procedures. To accomplish the objective, desired amount of PLA 6060D was dissolved in 20 mL of ethyl acetate and stirred continuously for 3 h at 100 °C by conduction on the hot plate. The PLA solution was emulsified using the oil-in-water (O/W, ethyl acetate/water) emulsion technique with non-ionic surfactants, precisely 50 μL of Tween 80 and Span 80. The prepared PLA solution was emulsified into 30 mL of ultrapure Milli-Q water using sonication for 30 s at 35 AMP. To achieve an equal weight of PLA and PUDs after drying, 7 mL of water-borne polyurethane was added to a polymeric emulsion consisting of 1% wt PLA, taking into account the weight of the PLA. The mixture was then sonicated for an additional minute. Subsequently, conductive inks containing MWCNTs, silver flakes, GNPs, and a combination of silver flakes with GNPs or MWCNTs were incorporated into the emulsion of PLA/PUDs. The fillers were added at a 30% wt concentration with respect to the dry polymer. The resulting mixture was then subjected to probe sonication for a duration of 3 min. The PLA concentration can be increased if an ink deposition technique requires higher-viscosity materials, such as screen printing or 3D printing. The ratio of binders to fillers can be adjusted to achieve the desired conductivity for a particular application, ranging from 0.01:100 to 50:100. The duration of tip sonication can vary between 1 and 30 min.

### Coating method

Because of the vast range of applications and needs in numerous markets, different coating processes are available. Coating is a procedure that transfers a layer of ink to a substrate by pouring, painting, spraying, casting, or smearing it over the surface^46,47^. Blade coating, spray coating, painting, slot-die coating, curtain coating, and slide coating are some coating processes. Spray coating techniques offer a high potential for large-scale production because they have no substrate size limitations and low polymer consumption, offering to replace existing spin coating methods^48^. Spray coating was used to deposit conductive ink onto the substrate to obtain a uniformly coated layer. The glass substrate was securely attached to a suitable metal plate, positioned at a 60° angle from the floor. An airbrush atomizer spray coater (VL Siphon feed, 0.73 mm nozzle internal diameter, Paasche airbrush, US) was used to spray 50 mL of inks, keeping its nozzle at a fixed distance of 17.5 cm from the substrate and a pressure of 2.5 bar. The atomizer was moved in various directions to ensure homogeneous spraying of the inks. The collected samples were given time overnight at ambient conditions to dry. Finally, to improve compactness, adhesion, and conductivity, the coated samples were annealed at various temperatures.

### Characterization

The coatings and the fabricated sensor were imaged by Scanning electron microscopy (SEM) using a JSM-7500FA (JEOL) equipped with a cold FEG, operated at 10 kV accelerating voltage. The energy-dispersive X-ray spectroscopy (EDS) analyses were conducted utilizing an Oxford X-Max system with an active area of 80 mm^2^. On a PANalytical Empyrean X-ray diffractometer equipped with a 1.8 kW CuK_α_ ceramic X-ray tube, PIXcel3D 22 mm^2^ area detector, and running at 45 kV and 40 mA, the X-ray diffraction (XRD) pattern of carbon-based active material and silver flakes were acquired. The diffraction pattern was generated at room temperature using a parallel-beam geometry and symmetric reflection mode over an angular range of 23°–70° with a step size of 0.05°. PANalytical’s High Score 4.1 software was utilized for phase detection. Raman spectroscopy measurements were conducted using a Renishaw microRaman Invia 1000 mounting a 50× objective, with an excitation wavelength of 633 nm and an incident power of 1 mW.

The PLA-based emulsions were imaged using a common optical microscope (Nikon microscope Eclipse 80i, Nikon Corp., Japan) connected to NIS Elements F Image Processing Software. The emulsions were lightly stirred in vials before analysis to ensure they were uniform. A drop of the emulsion was put on a microscope slide and then capped with a cover slip. More than ten pictures were taken for each type of emulsion. The PLA-based solution, emulsion, and conductive ink were characterized for average droplet size by the dynamic light scattering method (DLS, Malvern Zetasizer Nano ZS) working at 632.8 nm and 25 °C, with a He/Ne laser.

Infrared spectra were collected with a Fourier transform infrared spectrometer (Equinox 70 FT-IR, Bruker), with MIRacle attenuated total reflectance (ATR) (PIKE Technologies) using a diamond crystal. All spectra were recorded in the range between 4000 and 600 cm^−1^, by a resolution of 4 cm^−1^, accumulating 120 scans. Thermogravimetric analysis (TGA) was conducted on a TGA Q500 system (TA Instruments USA). The analyses were performed on ∼ 4.00 mg samples weighed in platinum pans and heated from 30 to 800 °C with a heating rate of 10 °C/min under nitrogen at a constant flow rate of 50 mL/min. Optical profilometer Zeta-20 by ZETA, with a z resolution of 10 nm, was used for surface mapping. A contact angle goniometer (OCA-20 DataPhysics, Germany) was used to measure the static water contact angles (WCAs) of the top layer of ink at room temperature. With the assistance of built-in software, 5 μL of deionized water was dropped onto the samples’ surfaces, and the contact angle was determined from the side view. Five measurements were made for each sample to guarantee the results’ reproducibility.

DMTA measurements in a Q800 (TA Instruments, USA) have analyzed thermomechanical sensitivity and viscoelastic behavior. A temperature ramp has been performed, from 30 to 150 °C, at a constant frequency (5 Hz) with a heating rate of 5 °C/min and for a displacement amplitude of 100 μm. Frequency sweeps have been conducted at temperatures ranging from the glassy plateau to the beginning of the α-relaxation.

Differential scanning calorimetry (DSC) was performed using Discovery DSC 250 TA Instruments. The measurements were carried out at heating and cooling rates of 20 °C/min from − 90 to 150 °C, with nitrogen as a cell purge gas (50 mL/min). In order to erase the thermal history, two heating runs separated by a cooling were made and the glass transition temperatures were deduced from the second heating. The measurements were conducted solely on the coating peeled off the glass substrate.

A manual probe station by Signatone 1160 (Microworld, France) provided direct currents and voltages. The IV (current–voltage) data were logged with the help of a Keithley 2612A system source meter (Tektronix, Inc. USA) coupled to the probe station. For each sample, five measurements at different points were carried out. Before the measurements, highly conductive silver paste (RS silver conductive paint, resistivity ∼ 0.001 Ω/cm) was applied at the opposite sides of the sample (around 1 cm apart) to minimize contact resistance. A bias voltage between − 2 and + 2 V is used to collect the IV data.

Mechanical properties of the coated sensors were studied with an Instron 3365 dual-column tabletop universal testing system on dog-bone samples with 25 mm length and 4 mm width. Tests were conducted with a 5 mm/min displacement rate until failure of the sample. All measurements were performed on three different specimens.

## Results and discussion

### Characterization of nanoparticle-surfactant-polymer stabilized emulsions

**Figure 1:**
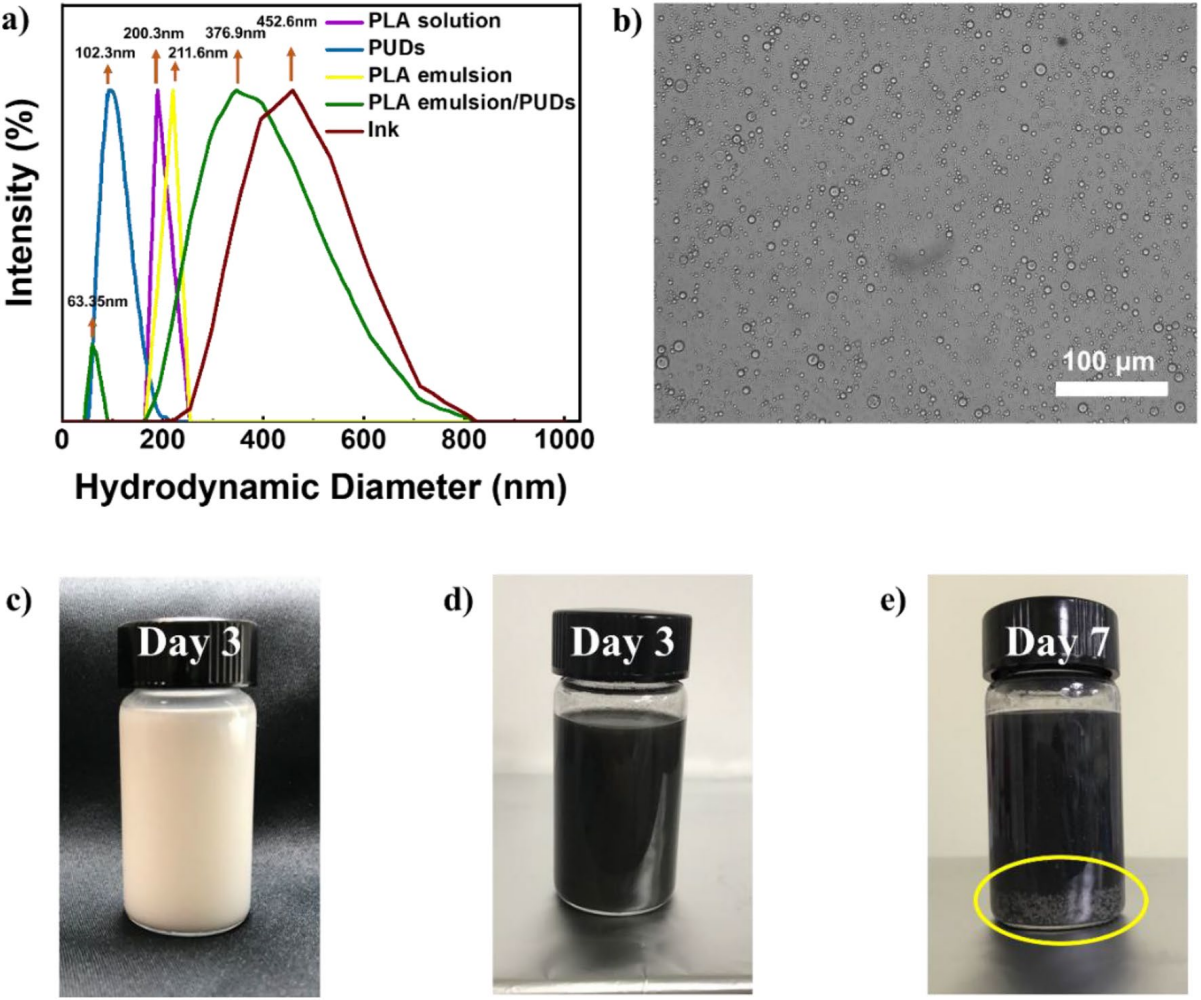
(**a**) DLS analysis specifies droplet size distribution of solution, emulsions, and the conductive ink. (**b**) Optical microscope image of PLA emulsion/PUDs. Photographs of (**c**) PLA emulsion/PUDs after 3 days, (**d**) ink after 3 days, (**e**) agglomerates of Ag flakes inside the ink after 7 days.

Various factors, including the storage duration and the emulsifying agent’s characteristics, can influence emulsions’ stability. Following a comprehensive screening procedure, it has been ascertained that a blend of surfactants from the Span 80 and Tween 80 series is efficacious in attaining the intended stability^49^. To evaluate the stability of the system, the mean droplet size of the emulsion was monitored for six hours (see Fig. 1a). For this measurement, all the solutions, aqueous dispersions, and emulsions were diluted to 0.1 mg/mL, and each sample was measured three times directly after the preparation and by the end of six hours. The droplet size distribution of PLA emulsion (in yellow) slightly increased from 0 min to 6 h and reached a fixed plateau value. This may be attributed to the reconnection of PLA nanoparticles inside the emulsion^49^. Further, adding PUDs (in green) to the PLA emulsion resulted in a significant increase in the mean droplet size of the emulsions. This may be due to the polydispersity of the emulsion droplets. Switching from the transparent PLA emulsion to the milky PLA emulsion/PUDs can be explained by the fact that the hydrophilic groups in the water-based polyurethane polymer chains of the continuous phase in the PLA emulsion, such as the carboxyl group or ammonium salt, are interconnected through physical connections^50^. Emulsions can be visualized through optical microscopy. The attainment of emulsions with small and uniform droplets (see Fig. 1b) that can impede the breakdown process is possible through the appropriate selection of emulsifiers. Conversely, unstable emulsions are characterized by dispersed droplets that have the propensity to coalesce and form larger droplets, commonly known as floccules. During the flocculation process, the merging of individual droplets increases the creaming degree observed in emulsions^51,52^. The average droplet size was 452.6 nm when the mixture of PLA emulsion/PUDs was combined with conductive fillers (CNTs and silver flakes) to create the conductive ink, and it remained remarkably stable. The excellent dispersion of conductive fillers in the emulsion can contribute to stabilizing the Pickering emulsion. The addition of CNTs and silver flakes to the emulsion can improve the stability of the emulsion, reduce droplet coalescence, and lower the interfacial tension between the two immiscible liquids. The hydrophobic nature of the ethyl acetate phase and the hydrophilic nature of the water phase create an energetically unfavorable interface in which the adsorption of conductive particles can stabilize. The conductive particulates can act as a surfactant, providing a strong and stable interface between the water and ethyl acetate phases. This surfactant effect is due to the high aspect ratio and large surface area of the conductive particles, which allows them to adsorb at the interface and form a stable barrier against droplet coalescence. Due to the unchangeable adsorption of solid particles at the interfaces of two immiscible liquids, the Pickering emulsion system exhibits significantly higher deformation resistance than conventional surfactant-stabilized emulsions. As a result, the mixture of PLA emulsion/PUDs combined with conductive fillers has improved stability and shelf life^53–55^.

In addition, Fig. 1c,d show that the ink has excellent stability during storage at ambient conditions for three days without phase separation. Nevertheless, it was noted that after a period of five days, the ink exhibited the occurrence of aggregation, leading to the subsequent sedimentation of silver flakes (see Fig. 1e). The results of this study indicate that the primary factor contributing to the low stability of silver-based conductive inks is the aggregation of silver flakes. This finding aligns with the current state of the art in the field^56^. Although nano-sized particles settle quite slowly and nanoparticles’ Brownian motion can counteract gravity’s effect, these nanoparticles still aggregate because of the attractive van der Waals forces between them. When particles collide and aggregate, because sedimentation velocity is proportional to the square of particle diameter, these agglomerates of larger size settle much faster and prevent the printability of silver-based conductive inks^57^. Hamaker constants can evaluate the attractive van der Waals forces between materials. For metals, their Hamaker constants are relatively high compared to common solvents. Therefore, silver suspension cannot maintain a well-dispersed state for long due to the attractive force between them^58,59^.

### Conductive coating characterization

**Figure 2:**
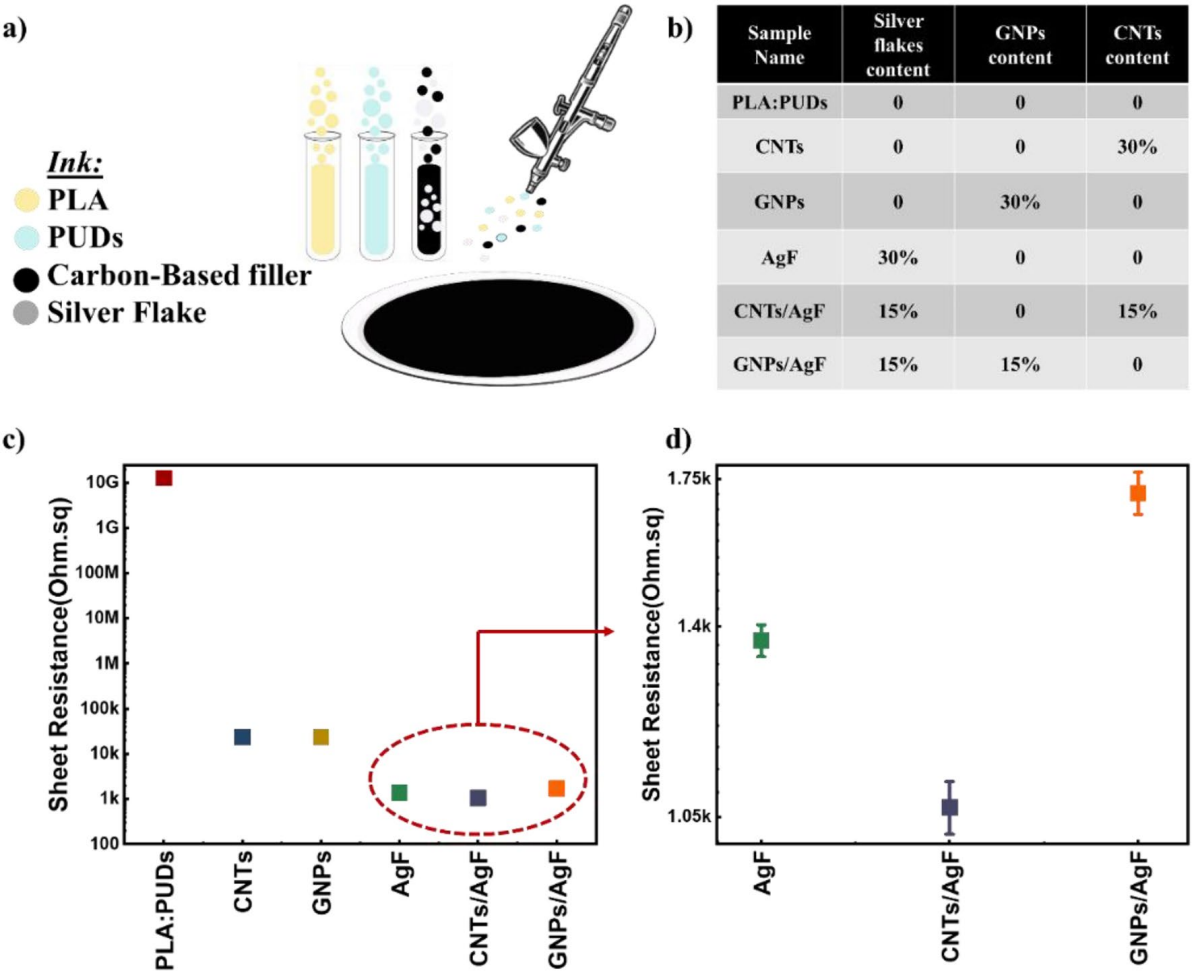
(**a**) Schematic representation of ink formulations and coating on paper substrate. (**b**) Conductive filler contents inside the inks compared to dry polymer basis. (**c**) Sheet resistance analyses for all the coated samples. (**d**) Similar magnification of sheet resistance measurement for the samples coated with hybrid formulated inks.

Figure 2a illustrates a schematic representation of ink formulations containing various conductive fillers. Spray coating methods are used to apply inks onto a paper substrate. Figure 2b summarizes the conductive filler content in the inks compared to the dry polymer basis. Figure 2c demonstrates that by combining silver flakes (σ = 6.107 S/m)^60^ and CNTs (σ = 1.104–2.105 S/m)^61,62^, sheet resistance decreased dramatically compared to silver flakes ink or ink made from a mixture of GNPs and silver flakes. This is due to the higher aspect ratio of CNTs compared to GNPs, which enhances bridging between adjacent silver flakes and accelerates electron transfer^63,64^. Moreover, by utilizing highly conductive hybrid CNTs and silver flake inks, good conductivity can be achieved with a lower silver content than conventional silver-filled inks. Figure 2d illustrates the comparable magnification of sheet resistance measurement for the hybrid formulated inks.

### Thermal treatment and its influence on the coating’s microstructure and conductivity

#### Choice of the thermal treatment temperature

**Figure 3:**
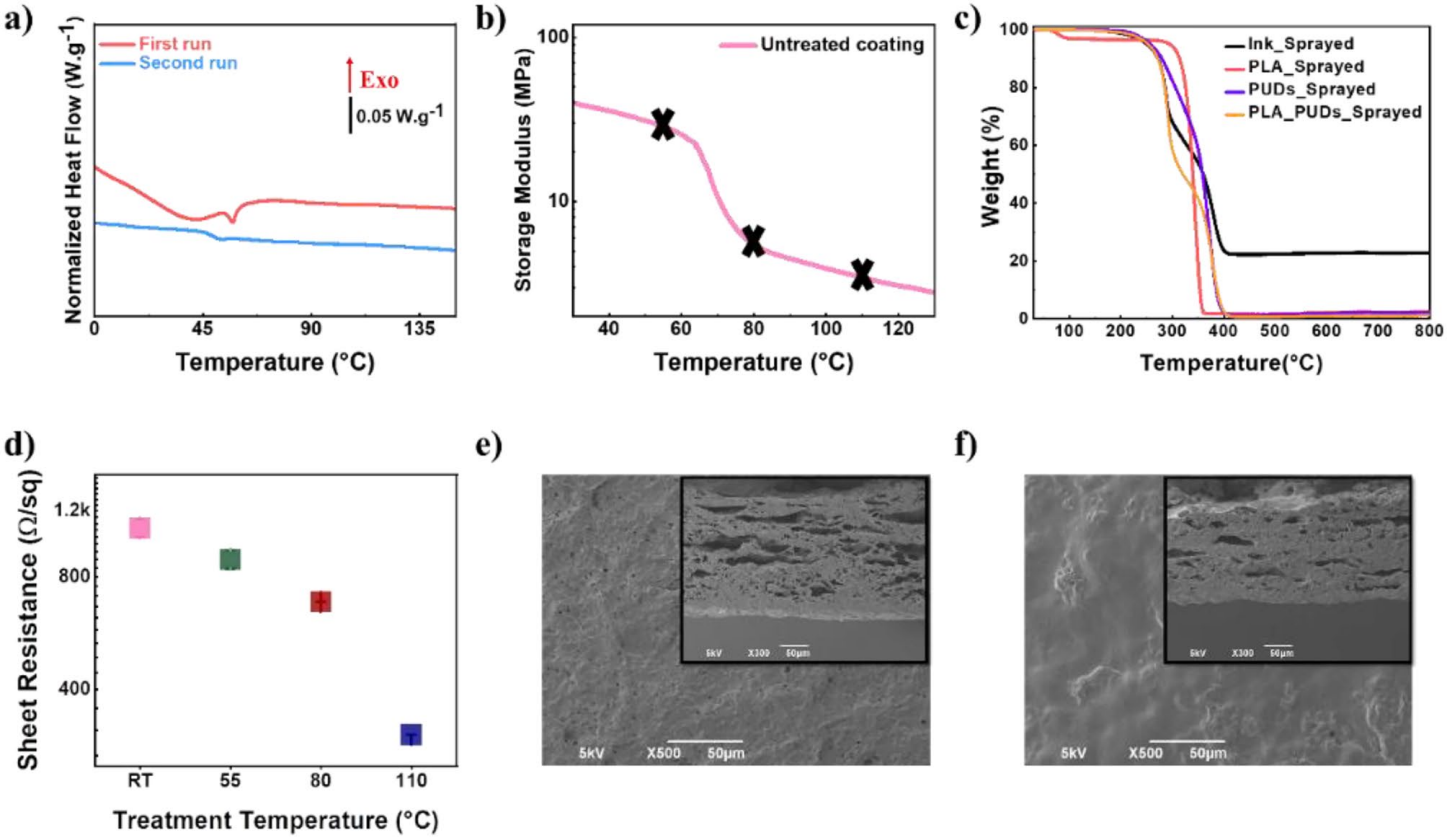
(**a**) Differential scanning calorimetry (DSC) measurement at 5 °C/min with heating runs ranging from 0 to 150 °C. (**b**) DMTA measurement of the untreated coating. The black crosses on the curve represent the three selected temperatures for the thermal treatment. (**c**) Thermogravimetric analysis (TGA) graph of the conductive coating and its ingredients. (**d**) Sheet resistance values of the untreated coating (RT) and the coating that has been thermally treated at 55, 80, and 110 °C for 2 h. Surface morphology of the coating observed by SEM (**e**) before and (**f**) after thermal treatment at 110 °C. The insets in e and f give cross-section views of the corresponding SEM images.

Thermal treatment was chosen as a post-treatment technique to improve the conductivity of the coatings further. Annealing over the glass transition temperature (Tg) of the polymer used as a binder can rearrange the chains in more closely packed configurations, can help to remove residual solvents, volatiles, or other impurities and cause the particles to pack more closely together, thereby increasing the density of the coating. In addition, this densification can lead to an increase in the conductivity of the coating, as it can reduce the number of voids or defects that can hinder the flow of electrical current^65,66^. In fact, to produce microstructural changes, the selected temperatures must be close to or higher than the coating’s glass transition temperature so that the polymer chains can move globally and cooperatively^67^. Differential Scanning Calorimetry (DSC) measurement of the coating was performed to determine multiple temperatures for thermal treatment. Figure 3a depicts the DSC curves of the free-standing coating. First, the coating is heated (the red curve), then quenched (not shown), and then heated again (the blue curve). The first stage of the protocol involves erasing the material’s thermal history, while the second involves the actual measurement^68,69^. According to the first run signal, the coating has a complex network. The baseline is hard to detect, as thermal dilatation follows the glass transition. During the glass transition, PLA releases enthalpy^70^. To avoid ambiguity, the findings were derived from the second heating run. With a heating rate of 5 °C/min, the coating’s glass transition temperature is around 49 °C.

In the second step, a DMA measurement was performed to better understand the evolution of rigidity with temperature. According to this measurement, three temperatures above the glass transition temperature have been chosen to perform the thermal treatments: 55, 80, and 110 °C, as it is assumed that they belong in a temperature range where polymer chains have mobility^70^ and can trigger microstructural changes that will improve the material’s conductivity, as demonstrated in previous studies^71,72^. In Fig. 3b, the black crosses on the storage modulus of the untreated coating (pink curve) represent the three temperatures selected for the thermal treatment (55, 80, and 110 °C). As can be seen, the three typical zones representing the polymer viscoelastic behavior are observable: the glassy plateau, up to around 60 °C, where chains have low mobility, followed by the α-relaxation which is noticeable by a drastic decrease in the rigidity. This relaxation leads to the rubbery plateau, where the rigidity is low because the chains exhibit high mobility. According to this graph, it can be expected that the thermal treatment at 110 °C allows the chains of the polymers to have higher mobility (middle of the rubbery plateau) in comparison with a treatment performed at 55 °C (beginning of the α-relaxation) and 80 °C (end of the α-relaxation). As the chains can quickly move at 110 °C, the coating can rearrange itself while improving the connections between the different conductive particles (CNTs and silver nanoparticles)^73^. The α-relaxation temperatures of the untreated coating are close (around 68 °C), in agreement with what is commonly reported for PLA, depending on the methods of measurement. The presence of the fillers is also responsible for the reduced mobility of the polymer chains, so an α-relaxation temperature is higher than the one of a pure PLA^74,75^. Finally, according to the thermogravimetric analysis (TGA) (see Fig. 3c, more details in Fig. S1), the coating did not show decomposition to volatile products in the temperatures of its annealing treatment.

#### Influence of thermal treatment on the electrical properties of the coating

The influence of the thermal treatment on the sheet resistance is noticeable, as it was also reported by other groups for PMMA filled with CNTs, polymer blends filled with carbon black, ladder polymers (BLL), PEDOT: PSS, or PP filled with CNTs^76–81^. In this work, the free-stand coatings have been treated at the selected temperatures for two hours. The efficiency of the thermal treatment has been evaluated for each temperature through sheet resistance measurement. Figure 3d depicts the sheet resistance measured before (labeled as RT) and after the thermal treatment for 2 h at 55, 80 and 110 °C. After the treatment at 55 °C, a slight decrease in the sheet resistance value was visible compared to the untreated coating, but the improvement remained low. After two hours at 80 °C, the sheet resistance was decreased by 1.5 times compared to untreated coating. The best result was obtained with the highest temperature used (110 °C), where the sheet resistance decreased by about 3.5 times and reached the value of 302.15 ± 2.23 Ω/sq. Additionally, in Fig. S2a, it is noticeable that the thermal treatment has induced no changes in the glass transition temperature compared to the uncured coating. Because there are no differences in the glass transition temperature for all temperatures tested compared to the untreated coating, it can be proposed that the electrical conductivity improvement is due to better filler connections and structural re-ordering^80,81^. Moreover, on the isothermal graphs (Fig. S2b), it is visible that the heat flow remains constant during all the measurements. It means that no crystallization occurred during this step, and findings from XRD measurements are consistent with these results (Fig. S2c). Based on micro Raman analysis (Fig. S2d), the typical bands D, G, and 2D can be clearly identified without any shifts between the bands of the polymer matrices before and after thermal treatment. It can be concluded that since the thermal treatment directly influences filler distribution, the thermal treatment for 2 h at 110 °C is the most effective way to trigger microstructural rearrangements that decrease the sheet resistance of the conductive coating. In other words, the thermal treatment at 110 °C has allowed microstructural changes in the coating, improving the connections between the conductive nanoparticles^81^. Figure 3e,f demonstrate the coatings’ surface with insets cross-sectional view of corresponding images for the coating prior to thermal treatment and after thermal treatment for 2 h. As seen in Fig. 3f, the morphology of the coatings slightly changed during the annealing step. The coatings’ surface turned from relatively rough with many voids into a smoother one, after thermal treatment at 110 °C. Increasing the annealing temperature from 55 to 110 °C (Fig. S3) made the material denser, as shown by a minor change in thickness (seen via cross-sectional observation); this hypothesis was validated by measuring the thickness. It is thought that some of the insulating impurities that remained in the coatings gradually evaporated off upon annealing, causing the coating to get more compact, and fillers came closer and improved the electrical conductivity.

#### Influence of isothermal treatment at 110 °C on the electrical properties of the coating

**Figure 4:**
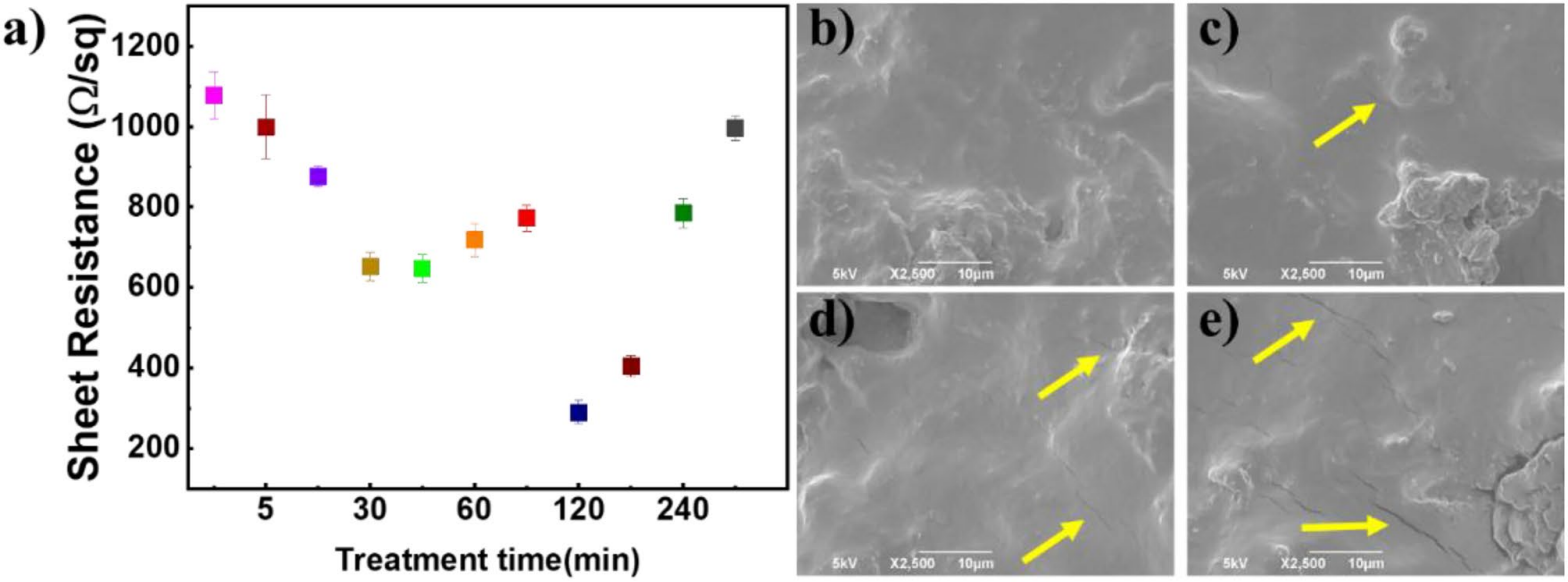
(**a**) Sheet resistance value for an isothermally treated sample at 110 °C from 0 to 300 min. SEM images taken after the isothermal treatment for (**b**) 120 min. (**c**) 180 min. (**d**) 240 min and (**e**) 300 min.

In general, exposure to higher temperatures, especially for longer durations, can change conductive coatings’ electrical and morphological properties^67,82,83^. To evaluate whether an increase or decrease in the time spent on prolonged treatment at the selected temperature can improve the electrical properties of the coating, isothermal treatment at 110 °C for varying time lengths was studied separately. Figure 4a depicts the sheet resistance evolution of the free-standing coating as a function of treatment time. With increasing time, the sheet resistance decreases, reaching a minimum of around 300 Ω/sq after 120 min. From 180 to 300 min at 110 °C, the sheet resistance increases slowly. As expected, with SEM analyses at high magnification, evidence of cracks was detected. After 120 min at 110 °C (Fig. 4b), no cracks were visible on the coating surface, while when the thermal treatment time was increased, cracks appeared and became numerous and deeper (see Fig. 4c–e). This impact seems time-dependent, and after 120 min of exposure, the crack’s depth, length, and width continued to increase. The formation of cracks causes defects in the electrical network, increasing the samples’ sheet resistance^49,84^. The crack formation can occur due to the thermal stresses generated during the thermal treatment at 110 °C. The formation and spread of cracks can be influenced by the coating material’s thermal expansion coefficient, conductive additives, temperature, and time^85–87^.

### Biochemical oxygen demand (BOD) in seawater

**Figure 5:**
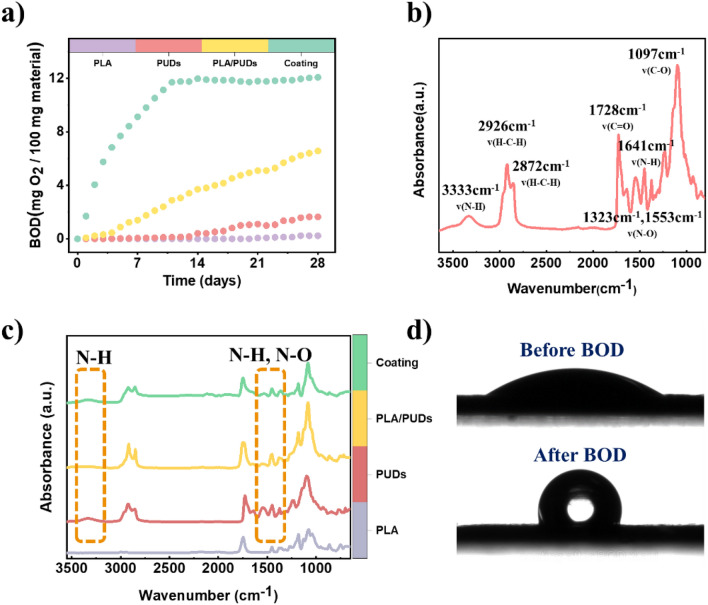
(**a**) Biochemical oxygen demand (BOD) of free-standing coating treated at 110 °C and its components in seawater. (**b**) SEM images of PLA/PUDs film. (**c**) Surface wettability measurement before and after BOD.

In case the conductive coating is accidentally released into the environment, it can undergo biodegradation, reducing waste material accumulation. This has been validated by examining biochemical oxygen demand (BOD), as depicted in Fig. 5a. When comparing the starting materials of polylactic acid (PLA) and polyurethane dispersions (PUDs) to a blend of PLA/ and PUDs and to the coating, it is seen that the resulting coating exhibits the greatest degree of biodegradability in seawater. All materials start exhibiting biodegradation in seawater within a short period of 3–4 days, and after approximately one month, the biochemical oxygen demand (BOD) levels for PLA/PUDs and coating reach values of 6.6 and 12.07 mg O_2_/100 mg material, respectively. In contrast, the biodegradation rate of the PUDs is significantly lower, with a recorded value of around 1.6 mg O_2_/100 mg material, following one month of biodegradation. The enhanced biodegradability of the coating, when compared to its initial constituents, can be attributed to many factors in the aquatic environment, which make the material more accessible to microorganisms.

The degradation rate of polylactic acid, when it is blended with polyurethane dispersions, is higher than that of PLA or PUDs alone. This observation can be supported by analyzing the FTIR spectra (Fig. 5b,c). The results of the examination using FTIR spectroscopy verified the existence of nitrogen in the PUDs, the mix of PLA and PUDs, as well as the coating material. Microorganisms necessitate nitrogen for their development and reproductive processes. The inclusion of nitrogen in PUDs and mixtures may augment their attractiveness as a nutritional resource for these microorganisms^88^. Moreover, when PUDs are blended with PLA, initially, there is an increase in the surface area, as it can be seen that a stable and immiscible blend can be formed with a high level of porosity (refer to Fig. S4) that causes a larger surface area. The increased surface area allows more microorganisms to attach to and break down the PLA/PUDs blend, resulting in faster biodegradation. Furthermore, increased hydrophilicity occurred by increasing the wettability of the blend surface. PLA possesses hydrophobic properties, indicating its resistance to water absorption. When polyurethane dispersions (PUDs) are mixed with polylactic acid (PLA), the blend becomes more water-friendly (Fig. S4), making it more likely to soak up water. Water absorption can create an optimal environment for microorganisms to thrive and decompose the PLA. It is evident that when PLA, PUDs, and fillers are blended together, they create a more hydrophilic structure (Fig. 5d) and water contact angle reach to 32.8° ± 0.3° in the coating. The increased hydrophilicity enhances water diffusion and consequently leads to a higher degradation rate of the coating. The coating’s hydrophobicity after BOD, as indicated by a water contact angle of 94.9° ± 1.1°, can be attributed to the coating’s increased surface roughness. The Zeta profilometer captured images demonstrate that the surface roughness of the coating increases when it is exposed to penetrating sea salt inside seawater (Fig. S6). The presence of sea salt was later confirmed through SEM analyses, as shown in (Fig. S6c,d). Additionally, the combination of PUDs and PLA can be dominated by a synergistic effect, resulting in a blend that possesses properties surpassing each component’s individual contributions.

Nevertheless, if these were the only explanations, the BOD curve for coating would be expected to be in close proximity to or fall between the BOD curves of PLA/PUDs and PUDs. Therefore, it can be inferred that additional factors contribute to the observed differences. Electroactive bacteria (EAB) are natural microorganisms, primarily belonging to the Bacteria and Archaea domains, which inhabit various environments such as water, soil, and sediment^89^. These microbes possess the unique ability to engage in electrical interactions, either among themselves or with their extracellular surroundings. Several recent studies have documented electroactive bacteria’s presence in marine and freshwater environments^90–92^. In recent years, there has been a noticeable increase in the attention given to electrogenic bacteria due to their potential utilization in environmentally friendly technologies focused on renewable energy and environmental stewardship^93^. Additionally, they can be employed for biodegradation or bio removal of specific pollutants^94–96^. The justification for the finding can be supported by inclusion of the conductive fillers in the coating, which can improve electron transfer between bacteria. This is particularly significant when comparing the higher biodegradation rate of the coating to that of PLA, PUDs, and PLA/PUDs. CNTs possess notable characteristics such as elevated electrical conductivity and a substantial surface area, which can effectively promote bacterial adherence and allow electron transfer^94,97^.

The process of biodegradation in aquatic environments presents more significant difficulties compared to industrial composting due to the lower temperatures and reduced concentration of microorganisms found in seawater. This finding demonstrates that using PUDs as the main ingredient enables the production of a higher proportion of biodegradable components compared to the exclusive usage of PLA as a binding agent^98–101^.

### Fabrication and sensing mechanism of a paper-based sensor

**Figure 6:**
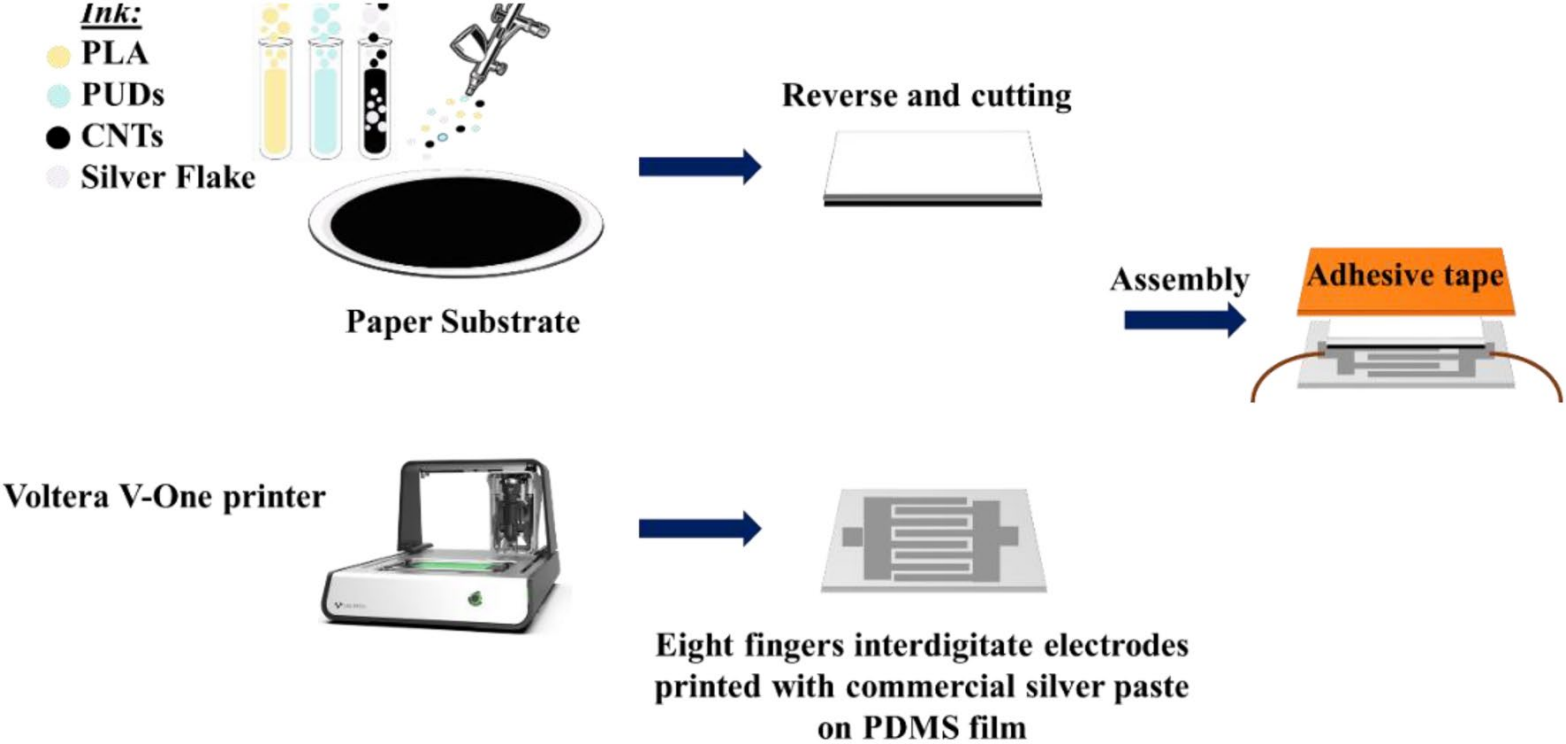
Schematic representation of the fabrication process of the pressure sensor.

Piezoresistive sensors are typically fabricated using materials that demonstrate alterations in electrical resistance in response to mechanical stress or strain^102^. Figure 6 illustrates a schematic depiction of the manufacturing process of a piezoresistive sensor using the previously developed conductive ink. The sensor’s construction involved implementing a three-layered structure. The uppermost layer of the structure was comprised of adhesive tape, fulfilling the role of a packaging layer. The intermediate layer was constructed using spray-coated paper with the previously developed conductive ink containing silver flakes and CNTs. This layer functions as the sensitive layer, exhibiting piezoresistive properties. A Voltera PCB printer was used to make the eight-finger interdigital electrodes on a flat polydimethylsiloxane (PDMS) substrate, which made up the bottom layer. This stratum serves as the output signal layer, functioning as the conductive plates.

To get the best print quality out of the Voltera V-One PCB printer, utilizing a glass-reinforced epoxy resin laminate substrate, commonly referred to as FR4, is essential. From this standpoint, a combination of liquid polydimethylsiloxane (PDMS) and a curing agent known as Sylgard 184 was blended at a weight ratio of 10:1. After being degassed in the vacuum desiccator, the PDMS was carefully poured onto the FR4 substrate. The FR4 substrate was then degassed and cured at 80 °C for 1 h to reach a final thickness of about 1 mm. Finally, using Voltera conductive silver ink, an eight-finger interdigitated layout was patterned on the FR4 substrate coated with PDMS film. When the printing layout had fully dried, the printed pattern separated from the FR4 substrate. The printed layout is an interdigitated pattern consisting of eight fingers, with overall dimensions of X = 21.0 mm and Y = 13.0 mm. The distance between two adjacent electrode fingers is approximately 500 μm. Figure S6 shows scanning electron microscopy (SEM) pictures depicting the printed pattern on the polydimethylsiloxane (PDMS) substrate. In order to optimize the signal recorded from the sensor, copper wire was affixed to both ends of the sensor.

#### Progressive compression test

**Figure 7:**
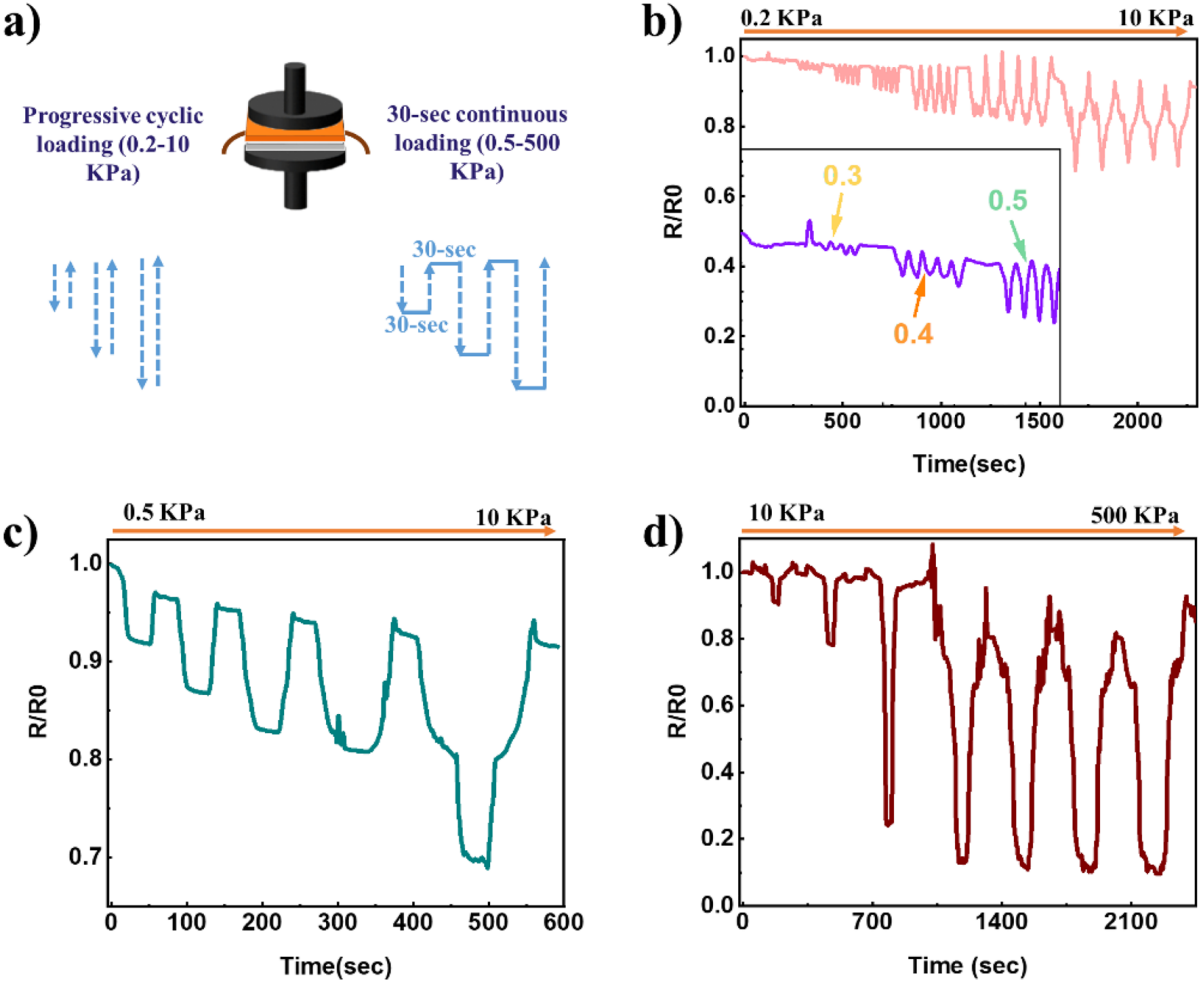
(**a**) Schematic representation of the progressive compression test. Sensor response during loading and unloading. (**b**) Cyclic low force load ranging from 0.2 to 10 kPa. (**c**) Step low force load ranging from 0.5 to 10 kPa. (**d**) Step high force load ranging from 10 to 500 kPa.

Since the sensor is designed to monitor repetitive pressure changes, it was subjected to a cyclic progressive compression test, and the effect of cyclic deformation on the resistance was investigated through Instron–Keithley coupled measurements. Generally, two phenomena occur in the system when the sensor is subjected to pressure: The destruction of some conductive networks as the inter-particle gap increases and the formation of new conductive networks as the conducting particle rearrange. The changes in electrical properties are determined by the dominance of one of these phenomena. Sensor performance was assessed in two scenarios. As these cycles frequently use either constant pressure or variable force. Given the applied electric voltage V and the measured current I, the resistance was calculated at each instant for each graph using the standard Ohm’s law and is plotted for independent sensors in Fig. 7a normalized to the initial resistance value R_0_.

According to analysis, adding external pressure reduces the distance between two neighboring conductive fillers, i.e., the average inter-particle gap is smaller, resulting in a lower sensor total resistance^103–105^.

In the first scenario (Fig. 7b), the constructed sensor was positioned between Instron clamping plates. A low-force load cell (10 N) was used to record precise pressure, which was gradually increased from 0.2 to 10 kPa (0.2, 0.3, 0.4, 0.5, 1, 2–5, and 10 kPa). Each step was repeated five times before increasing the pressure value. During the unloading, the pressure was reduced to 0.1 kPa with a 30-s relaxation period between two loading pressures. The sensor output is unchanged in the pressure range of less than 0.3 kPa, as highlighted in the inset magnification of Fig. 7b. Despite the increased applied pressure at each cycle, the current detection performance was stable and continuous, with no apparent signal loss when loading and unloading.

Looking at Fig. 7c, the second scenario involved putting the sensor through a pressure range from 0.5 up to 10 kPa. Each cycle consisted of 30 s of applying pressure, followed by 30 s of releasing it. After each cycle, the sensor demonstrated an excellent recovery from pressure when left to relax the stress, returning to almost the same resistance value compared to the initial R_0_. This was particularly noticeable at higher pressures ranging from 10 to 500 kPa (Fig. 7d), where the same behavior was observed. This drop in the sensor’s relative resistance after being loaded with pressure is most likely caused by a better arrangement of the fillers contained within the coating^74,106^. The cone-shaped structure of the graph changed when the applied force was more significant than 5 kPa. This can be explained by the fact that, when the sensor is subjected to pressure, the fillers inside the intermediate layer that serves as the sensitive layer rearrange themselves. In contrast, the output signal layer printed on the PDMS film slightly contrasts the applied force. When PDMS is subjected to a compressive load, it undergoes deformation, and its thickness decreases. The compression behavior of PDMS is affected by various factors, such as the magnitude and rate of the applied pressure and the duration of compression. Studies have shown that PDMS exhibits nonlinear compression behavior, as is evident in the hysteresis loop shown in Fig. S7, where the compressive stress–strain response is highly dependent on the level of deformation. At low levels of deformation, PDMS behaves elastically, and the stress–strain response is linear. However, as the deformation increases, PDMS exhibits a more nonlinear stress–strain response, and at very high levels of deformation, it can undergo permanent deformation^107,108^.

## Conclusion

This research primarily focuses on the advancement of environmentally friendly conductive inks, with the objective of diminishing dependence on hazardous solvents and investigating sustainable alternatives. In this study, conductive ink was formulated by adding silver flakes and multi-walled CNTs as conductive fillers to PLA/PUDs. In order to accomplish that, emulsification was employed, wherein a significant portion of the solvent was replaced with water. Dynamic light scattering and optical microscopy were used to analyze the emulsion and ink. The study’s findings revealed that the oil-in-water emulsion demonstrated a consistent distribution, including PLA, PUDs, and hybrid fillers. In addition, the emulsion exhibited long-term stability for a duration of several days. The inks were spray coated and subsequently heat treated to enhance their electrical conductivity of the formed coating. The sheet resistance exhibited a decrease following treatment at a temperature of 110 °C, ultimately reaching a minimum value of 300 Ω/sq after a duration of 120 min. However, with further increasing the treatment time, cracks formed and got more numerous and deeper. The presence of these fissures results in imperfections within the electrical network, leading to an elevation in sheet resistance.

The coating showed improved biodegradability in seawater, with respect to the individual components. This can be attributed to various causes, including the incorporation of nitrogen within its structure, which enhances the material’s susceptibility to degradation by microorganisms. Apart from this, the concurrent use of PUDs and PLA might be predominantly influenced by a synergistic phenomenon that facilitates the adhesion of bacteria and the transfer of electrons.

A piezoresistive sensor was fabricated by employing paper as a flexible substrate and coating it with conductive ink. The sensor featured a three-layered construction, including adhesive tape, conductive ink-coated paper, and interdigital electrodes printed on a PDMS substrate by the Voltera PCB printer. The results of a cyclic progressive compression test indicated that the sensor possesses a high-pressure sensitivity of 0.3 kPa and can operate within a range of 0.2 to 500 kPa. During the cyclic pressure test, a drop in relative resistance was observed, which can be attributed to the reduction in the distance between adjacent conductive fillers induced by external pressure.

### Supplementary Information

Supplementary Figures.

## Data Availability

The data that supports the findings of this study are available within the article and its supplementary materials. Additionally, upon reasonable request, further data may be made available by contacting the corresponding author at Maedeh.najafi@leibniz-inm.de.
