# Supplementary material for: Biodegradable polylactic acid emulsion ink based on carbon nanotubes and silver for printed pressure sensors
Source: Sci Rep. 2024 May 14;14:10988. doi: 10.1038/s41598-024-60315-z (PMC11094035; doi:10.1038/s41598-024-60315-z)
Supplement: Supplementary file 1 — Supplementary Figures. [file 41598_2024_60315_MOESM1_ESM.pdf]

Supporting Information

Biodegradable polylactic acid emulsion ink based on carbon nanotubes and silver for printed pressure sensors

Maedeh Najafi[a,b]*****, *Emilie Forestier*[a,c], *Milad Safarpour*[a], *Luca Ceseracciu*[d], *Arkadiusz Zych*[a], *Ahmad Bagheri*[e], *Laura Bertolacci*[a], *Athanassia Athanassiou*[a,]***, *Ilker Bayer*[a,]*

[a] Smart Materials, Istituto Italiano di Tecnologia, Via Morego 30, Genova 16163, Italy

[b] INM-Leibniz Institute for New Materials, Campus D2.2, 66123 Saarbrücken, Germany

[c] iCub Tech, Istituto Italiano di Tecnologia, via S. Quirico 9d, 16163 Genova, Italy

[d] Materials Characterization, Istituto Italiano di Tecnologia, Via Morego 30, Genova, 16163, Italy

[e] Graphene Labs, Istituto Italiano di Tecnologia, via Morego 30, 16163 Genoa, Italy

E-mail: Maedeh.Najafi@leibniz-inm.de, Athanassia.Athanassiou@iit.it, Ilker.Bayer@iit.it

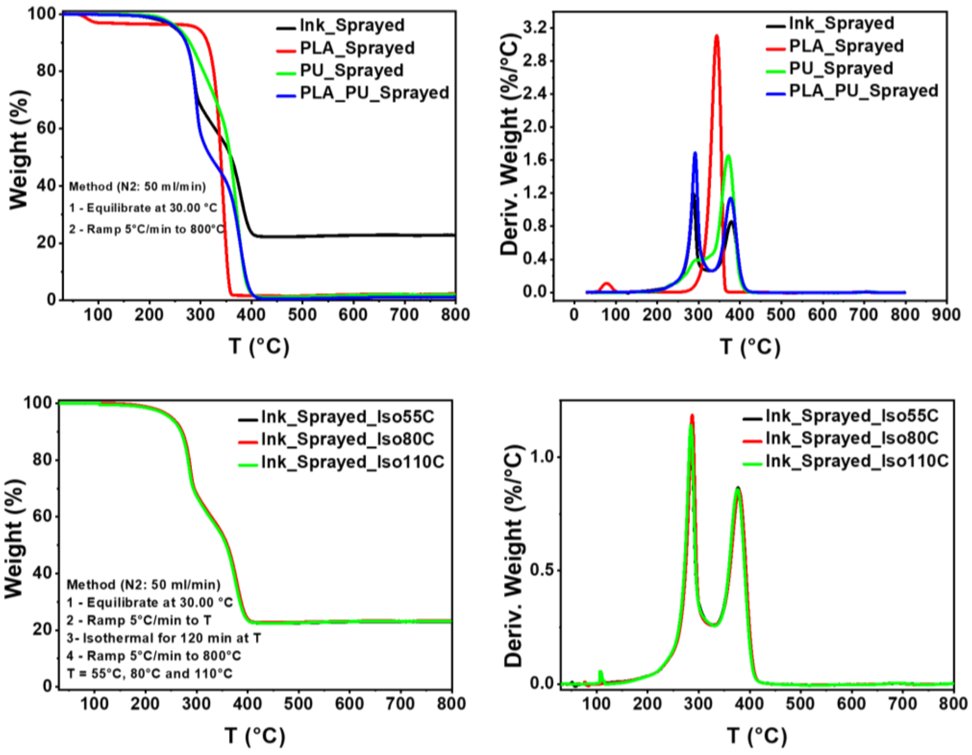

Figure S1. *Thermogravimetric Analysis (TGA) graph of the coating and its ingredients.*

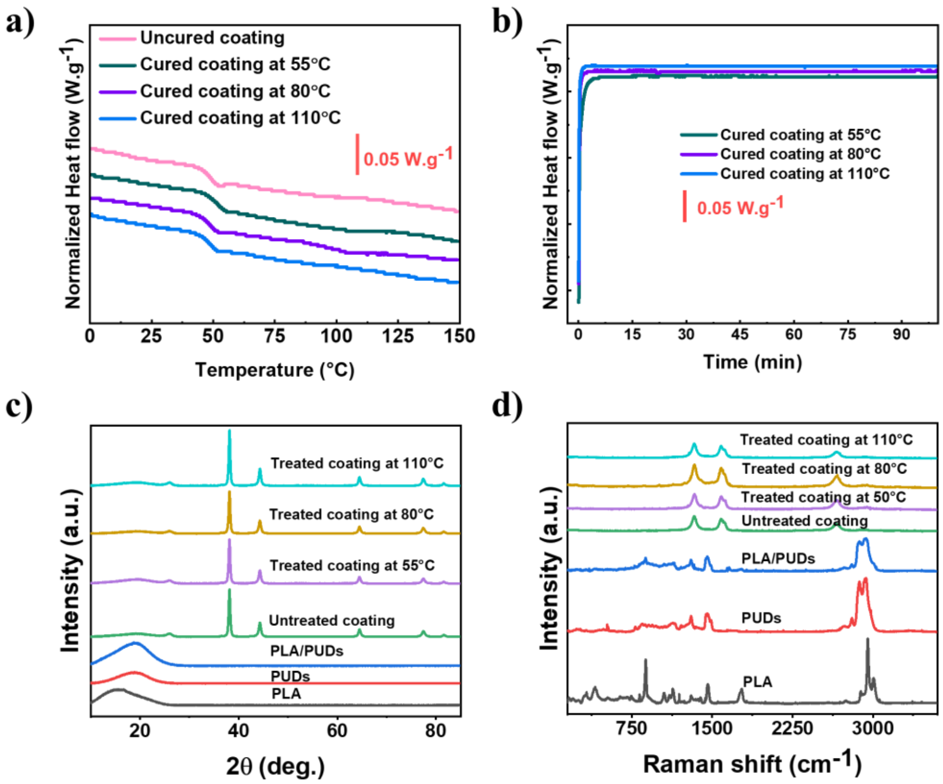

Figure S2. *a) Differential scanning calorimetry (DSC) measurement (second heating run) at 5°C/min for untreated and treated coating. b) Isothermal treatment performed during 100 minutes at 55 ºC, 80 ºC and 110 ºC .c) XRD measurements of the coating annealed at different temperatures (Reference code:98-005-3761). d) Micro Raman analysis for coating annealed at different temperatures.*

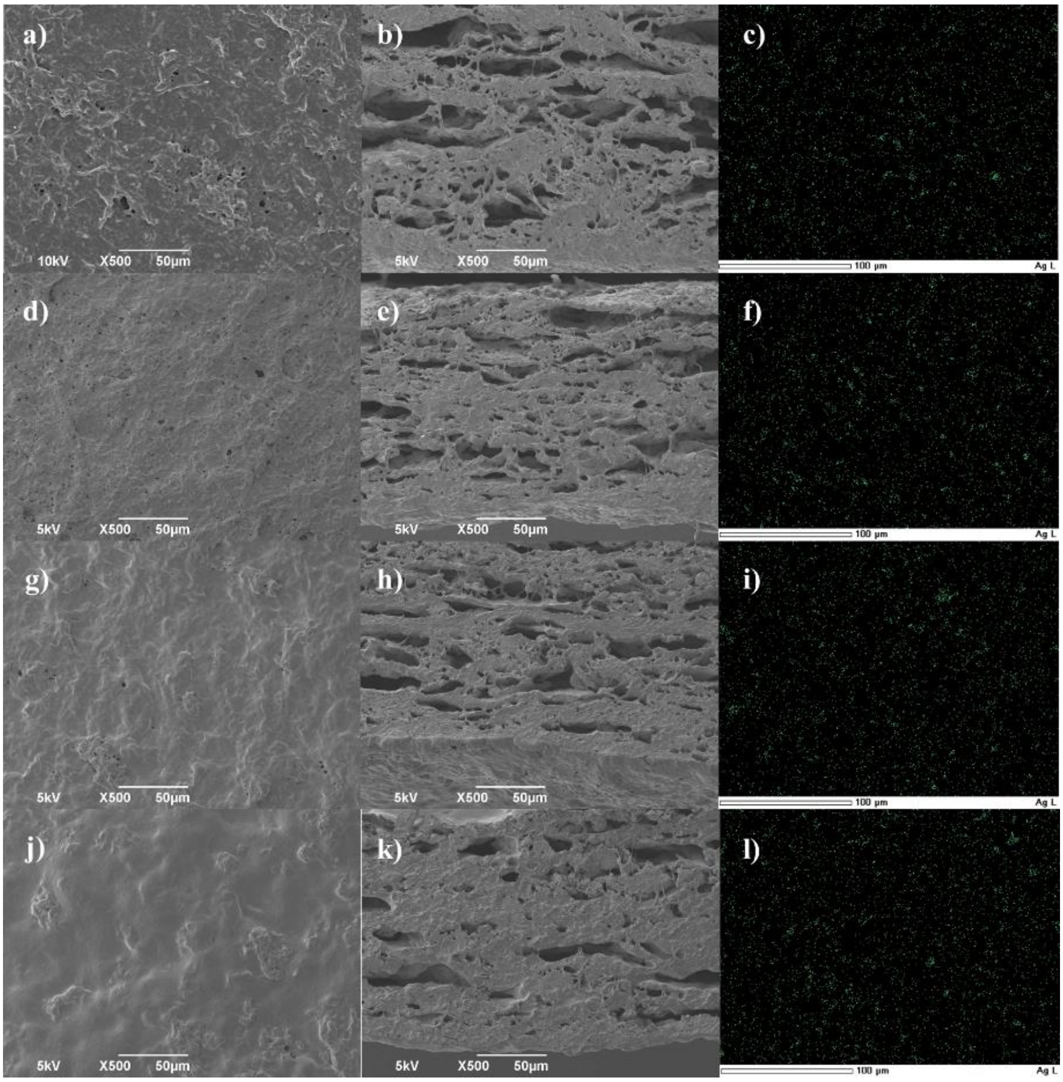

Figure S3. *SEM images and EDS analyses of the microstructural evolution of the coatings: a, b, and c) before the thermal treatment. After thermal treatment at different temperatures: d, e, and f) 55°C. g, h, and i) 80°C. j, k, and l) 110°C.*

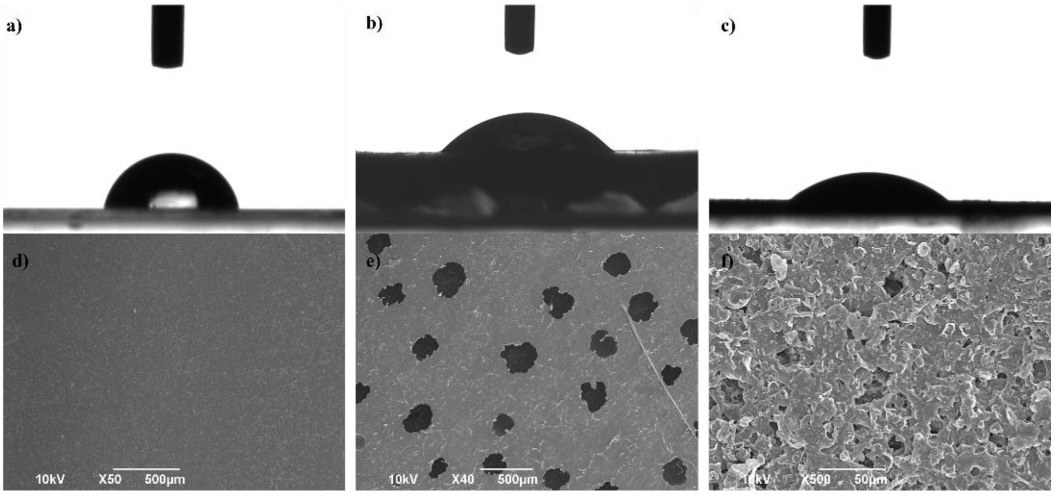

Figure S4. *Surface wettability measurement immediately after disposing of water on a) PLA. b) PUDs and c) PLA/PUDs. SEM images of the surface after drying in the ambient environment: d) PLAce) PUDs. f) PLA/PUDs.*

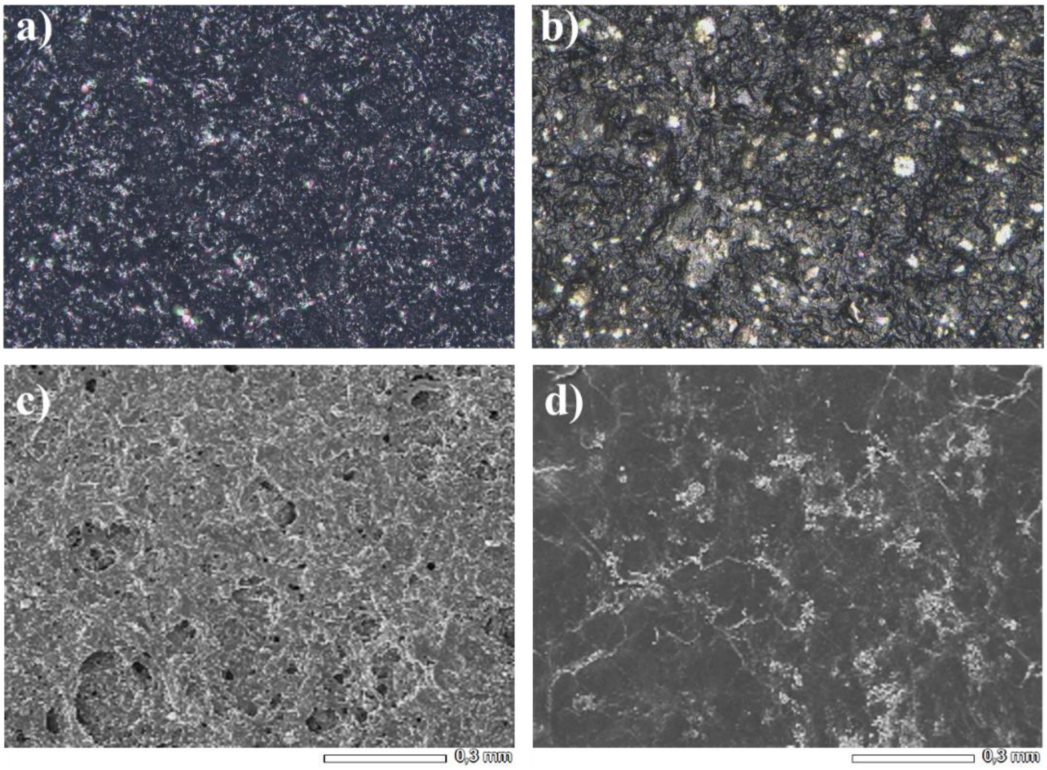

Figure S5. *Zeta profilometer images of the coating: a) before the BOD test. b) following the BOD test. SEM images of the coating's surface components: c) before the BOD test. d) following the BOD test.*

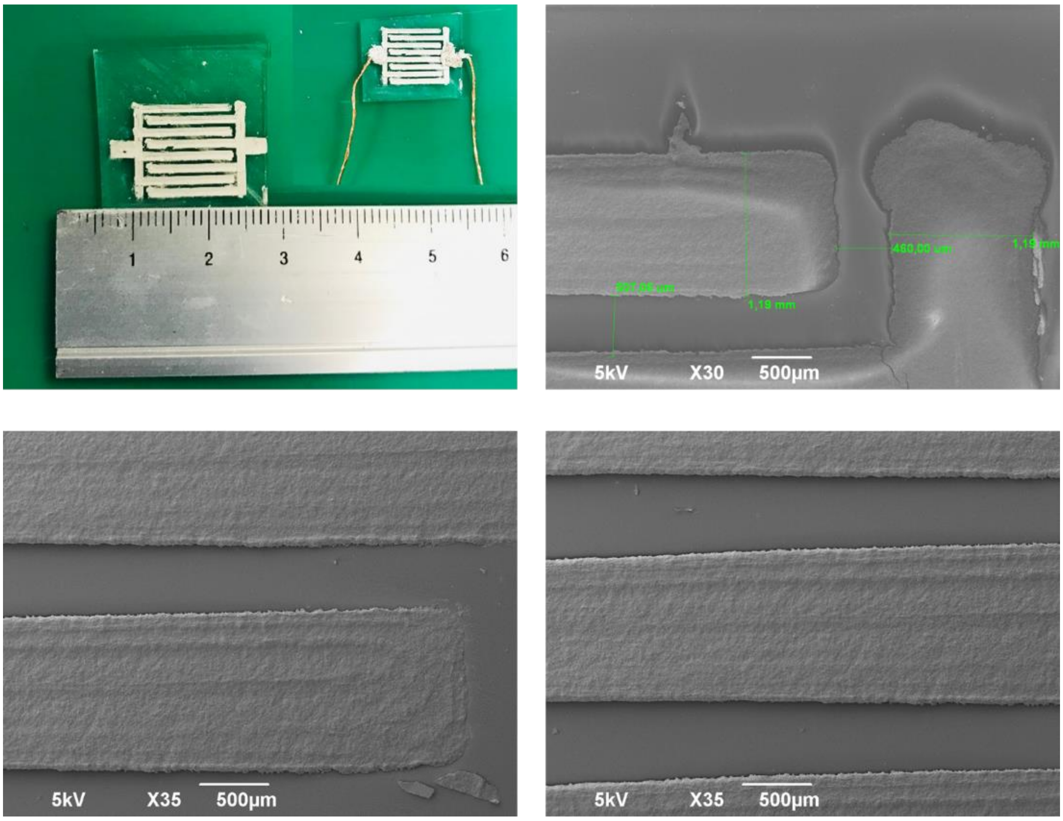

Figure S6. Printed layout on the PDMS substrate and following SEM images of it.

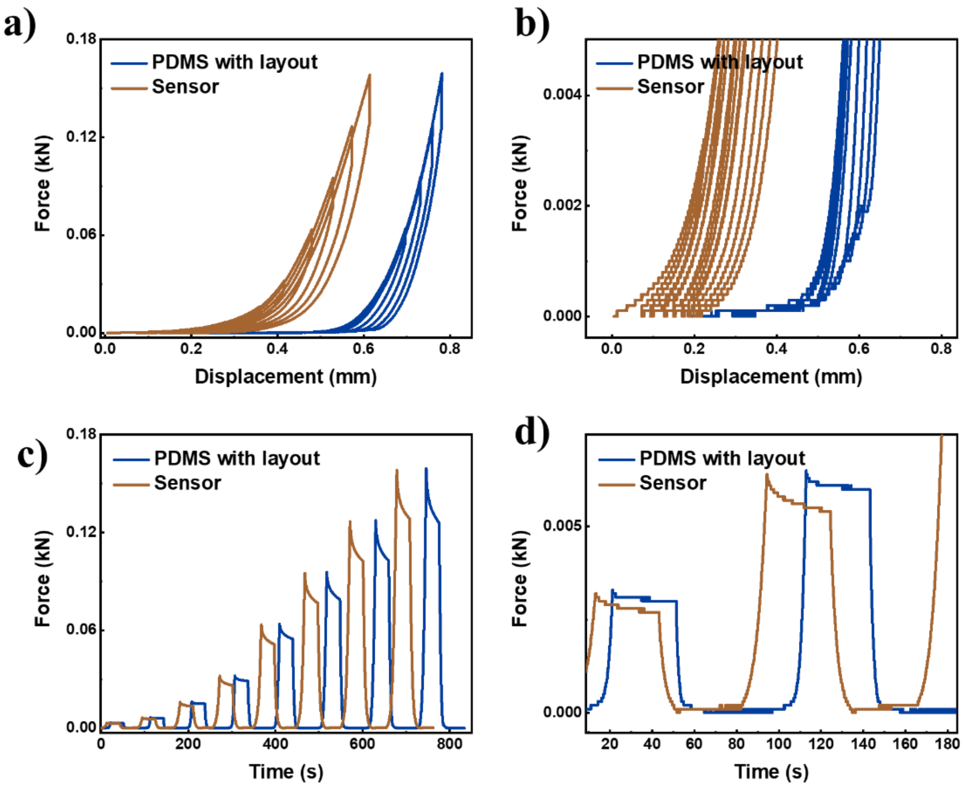

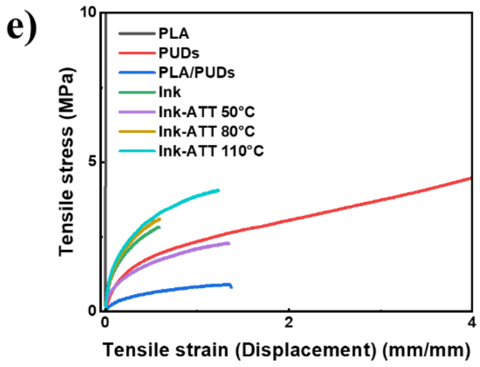

f)

sample	Young's Modulus (MPa)	SD
PLA	2544.43	678.43
PUDs	3.31	0.81
PLA/PUDs	2.87	0.14
Ink	13.96	1.65
Ink-ATT-50°C	16.86	0.81
Ink-ATT-80°C	27.98	1.32
Ink-ATT-110°C	28.89	1.11

Figure S7. a-d) Hysteresis loop for the sensor and PDMS printed with the layout. e-f) Mechanical properties of the ink and its ingredients
